# Efficacy of wearable devices detecting pulmonary congestion in heart failure: a systematic review and meta-analysis

**DOI:** 10.3389/fcvm.2025.1612545

**Published:** 2025-08-11

**Authors:** Cian P. Murray, Andrew P. Kenny, Niall J. O’Sullivan, Ross T. Murphy, James P. Curtain

**Affiliations:** Department of Cardiology, St. James’s Hospital, Dublin, Ireland

**Keywords:** heart failure, wearable devices, remote monitoring, pulmonary congestion, hospitalisations

## Abstract

**Introduction:**

Heart failure (HF) hospitalizations are prognostically significant. Implantable hemodynamic monitors detect early congestion but are invasive and costly, with no clear mortality benefit. Wearable devices offer a non-invasive alternative for monitoring congestion. This meta-analysis examines the efficacy of wearable devices in reducing HF hospitalizations and mortality compared to standard care.

**Methods:**

A systematic review and meta-analysis were conducted following PRISMA guidelines. PubMed, EMBASE, MEDLINE, and Cochrane databases were searched for trials comparing wearable device-guided care with standard HF treatment. Outcomes included hospitalisation for HF, worsening HF events (hospitalisation or emergency department visit for HF) and all-cause mortality. Total (first and recurrent) event meta-analyses were performed using random effect models.

**Results:**

Four studies met inclusion criteria, including 958 patients who were enrolled either at the time of or within 10 days of discharge from a hospitalization for HF. Wearable device-guided care resulted in a 41% reduction in hospitalisations for HF (RR: 0.59, 95% CI: 0.41–0.87, *p* = 0.007) and a 40% reduction in HF events (RR: 0.60, 95% CI: 0.42–0.86, *p* = 0.005) compared to standard care. All-cause mortality was reduced by 26% in the wearable monitoring arm (RR: 0.74, 95% CI: 0.55–0.99, *p* = 0.04). The composite outcome of HF hospitalization and mortality was 37% lower with wearable monitoring (RR: 0.63, 95% CI: 0.44–0.91, *p* = 0.04). Treatment for HF, guided by wearable devices that measure pulmonary congestion, reduced hospitalisations for HF and all-cause mortality in recently hospitalised patients.

**Conclusion:**

Wearable devices are a promising non-invasive strategy for managing high-risk patients, particularly when transitioning care from acute to community settings..

**Systematic Review Registration:**

https://www.crd.york.ac.uk/PROSPERO/view/CRD42024607770, identifier PROSPERO CRD42024607770.

## 1 Introduction

Hospitalisations for congestion are a hallmark of heart failure (HF) with each admission being of prognostic importance (1, 2). Implantable haemodynamic monitor (IHM) studies have demonstrated that subclinical alterations in physiology occur weeks before the development of overt clinical signs and symptoms that lead a patient to present to their care provider including a rise in intra-cardiopulmonary pressures and pulmonary congestion (3–5). Body weight is often monitored as an outpatient for the development of congestion but may not accurately predict decompensation (6, 7). IHMs such as pulmonary-artery sensors may reduce hospitalisations for HF but have yet to demonstrate a mortality benefit and are costly, requiring a dedicated invasive procedure (8). IHM-guided care received a modest class IIb recommendation in the 2021 European Society of Cardiology guidelines for HF (9). Wearable devices (wearables) offer a potential non-invasive alternative method of detecting pulmonary congestion and changes in physiological parameters (10, 11). Wearables could be applied readily by patients or carers with no requirement for an invasive implant and used by patients with HF across the range of ejection fraction (EF). In this meta-analysis, we examined the efficacy of these novel technologies that measure pulmonary congestion to reduce hospitalisations for HF, worsening HF events [hospitalisation for HF or emergency department (ED) visits for HF therapy] and mortality, compared with standard, unmonitored care.

## Methods

### Study design and search strategy

A systematic review of trials in patients with HF was performed, comparing wearable-guided care vs. standard treatment alone. This meta-analysis is registered on PROSPERO (CRD42024607770). The Preferred Reporting Items for Systematic Reviews and Meta-Analyses (PRISMA) guidelines were followed to conduct the literature search, data extraction and reporting (12). A PRISMA checklist is included in the Supplementary Appendix Table S1. Bias was assessed using the Revised Cochrane Risk-of-Bias Tool for Randomised Trials V.2 (Supplementary Appendix Table S2) (13). Literature searches were performed on several databases (PubMed, EMBASE, MEDLINE and Cochrane) with the last search performed on November 1st 2024, using the terms “(HF OR congestive HF OR Acute HF) AND (wearable device* OR remote monitor* OR wearable sensor* OR lung fluid monitor OR lung impedance OR remote dielectric sensing) AND (hospitaliz* OR hospitalis* OR rehospitalis* OR rehospitaliz* OR admission* OR readmission* OR re-admission OR Mortality OR ED Presentations)”. Hand-searches of reference lists from the identified articles were performed. No restriction was placed on study size, language or country of publication. Titles and abstracts were screened according to pre-specified inclusion criteria using the population, intervention, comparator, outcomes and study (PICOS) framework:

• Population: patients with HF across ranges of EF
• Intervention: treatment for HF guided by wearable monitors that detect pulmonary congestion
• Comparator: standard (unmonitored) care for HF
• Outcomes:
  ○ Hospitalisation for HF
  ○ Worsening HF events (Hospitalisation for HF or ED visit for HF management)
  ○ All-cause mortality
  ○ All-cause mortality and hospitalisation for HF
• Study design: randomised controlled trials or non-randomised studies with a concurrently enrolled control arm

Full text articles of original studies were included. The Cochrane Collaboration's screening and data extraction tool, Covidence, was utilised to streamline data extraction and storage. Two researchers (CPM and APK) independently performed the literature searches and data collection including study characteristics for eligibility, participant and event numbers. Results were compared and differences were resolved with consensus from a third author (JPC). All authors reviewed the analysis and contributed to drafting the report. Each of the included studies in this meta-analysis were conducted with local institutional ethical approval.

### Statistical analysis

The statistical analyses were performed using RevMan (version 5.4.1; Copenhagen: The Nordic Cochrane Centre, The Cochrane Collaboration, 2014). As the included studies examined three different devices across two decades a random-effect [DerSimonian and Laird (D + L)] model (14) was used so that differences in study design and cohorts would be accounted for within the analysis. *I*^2^ statistic for percentage heterogeneity was computed with corresponding *p* values (15). Forest plots graphically report the pooled effect size estimates, the degree of heterogeneity and the weighted contribution each study made to the analyses. A *p*-value less than 0.05 was considered statistically significant.

### Efficacy endpoints

The following clinical outcomes were examined according to study and device:

• Benefit of Microcor in Ambulatory Decompensated HF (BMAD) (16): (1) time-to-first hospitalisation for HF (2) time-to-first worsening HF event (hospitalisation for HF or ED visit for HF management) (3) all-cause mortality
• Bensimhon et al, Remote Dielectric Sensing system (ReDS) (Sensible Medical Innovations, Israel) (17): (1) total (first and recurrent) hospitalisations for HF
• Non-Invasive Lung IMPEDANCE-Guided Preemptive Treatment in Chronic Heart Failure Patients (IMPEDANCE-HF) (18): (1) total hospitalisations for HF (2) all-cause mortality
• Remote Dielectric Sensing Before and After Discharge in Patients With ADHF (ReDS-SAFE HF) (19): (1) total hospitalisations for HF (2) all-cause mortality

Numbers of clinical events and numbers of study patients in each study arm were extracted according to outcome. Risk ratios (RR) were calculated with 95% confidence intervals. Meta-analyses of the effect estimates were performed for (1) hospitalisation for HF (2) worsening HF event (hospitalisation for HF or ED visit for HF management) (3) all-cause mortality (4) hospitalisation for HF and all-cause mortality.

## Results

### Literature review and search results

3,865 articles were identified by searching electronic databases. After excluding 1,468 duplicates, the abstracts of 2,397 studies were assessed for potential inclusion. Full text review of 45 studies resulted in the identification of four published studies that met the inclusion criteria. These four studies were included in the analysis (16–19). Both 30-day and 90-day outcomes were reported in the ReDS trial with the 90-day results included in this meta-analysis. The search process and identification of relevant articles are summarised in a PRISMA flow chart (Supplementary Appendix Table S3).

### Study and investigational device characteristics

Four eligible studies were identified (Table 1). BMAD was an international, prospective concurrent control trial studying the Zoll Heart Failure Monitoring System (HFMS) (Zoll, Pittsburgh, USA). The Zoll system uses a novel radiofrequency sensor to estimate thoracic fluid content. The trial compared a control arm (BMAD-HF) with an intervention arm (BMAD-TX) over a 12-month follow-up period. In the BMAD-TX arm, device-collected data were used to guide clinical HF management.

**Table 1:** Studies of HF management guided by wearable devices that detect pulmonary congestion compared with standard, unmonitored care.

Author, Year	Design, country	Wearable device, measures, wear-time	Key inclusion criteria	Primary outcome	Secondary outcomes	Follow up (months)
Alvarez-Garcia et al. (19)	Randomised control trial *Single blind Multinational	ReDS Lung fluid content 45 s	HF hospitalisation at enrolment No LVEF inclusion criterion NT-proBNP ≥400 pg/L or BNP concentration of ≥100 pg/L BMI (kg/m^2^): >22, <39	Urgent outpatient visits for HF, HF hospitalisation, or all-cause mortality	Components of the primary outcome Length of hospitalisation stay Change in 6-min walking test Change in KCCQ questionnaire Change in NYHA functional class Clinical evidence of congestion at follow-up Change in natriuretic peptides	1
Boehmer et al. (16)	Concurrent-control clinical trial *Single blind Multinational	Zoll HFMS Thoracic fluid content, ECG, respiratory rate, activity, posture Continuous (24 h)	Recurrent HF hospitalisation • <10 days • <6 months No LVEF inclusion criterion	HF hospitalisation	All-cause mortality HF hospitalisation or ED visit for HF	12**
Bensimhon et al. (17)	Randomised control trial *Single blind USA	ReDS Lung fluid content 90 s	HF hospitalisation at enrolment No LVEF inclusion criterion BMI (kg/m^2^): >22, <38 BNP (pg/ml): >200	Lung fluid content	HF hospitalisation	3
Shochat et al. (18)	Randomised control trial *Single blind Israel	LI Lung impedance Wear-time n/r, in-office measurements	HF hospitalisation ≤12 months LVEF ≤35% NYHA class II–IV	HF hospitalisation	All-cause mortality	48 ± 32

BMI, body mass index; BNP, B-type natriuretic peptide; ECG, electrocardiogram; ED, emergency department; HF, heart failure; HFMS, Heart Failure Monitoring System; KCCQ, Kansas City Cardiomyopathy Questionnaire; LI, Lung Impedance; LVEF, left ventricular ejection fraction; NYHA, New York Heart Association; ReDS, remote dielectric sensing system.

* Investigators were aware of the monitor data in the treatment group but not the control group.

** 90 day outcomes were published.

Lung impedance (resistance) to an electric current passed across pulmonary tissue reduces as pulmonary congestion develops in people with HF (20–24). Thoracic impedance has been shown to correlate strongly with pulmonary capillary wedge pressure in invasive monitor studies (25). The IMPEDANCE-HF randomised controlled trial examined the effectiveness of the RS-205 wearable lung impedance (LI) monitor (RS Medical Monitoring, Jerusalem, Israel), to estimate pulmonary congestion and guide HF treatment compared with unmonitored HF care over a mean follow up period of 48 months (26).

Both of the studies by Bensimhon et al. and Alvarez-Garcia et al. were randomised trials examining the Remote Dielectric Sensing (ReDS) System (Sensible Medical Innovations, Israel). The ReDS device is a wearable vest that quantifies the percentage of lung fluid compared to lung volume by analysing the dielectric coefficient of the lung between the vest sensors (27, 28). The ReDS trials included in this meta-analysis examined clinical outcomes in people who received treatment guided by ReDS detected pulmonary congestion compared with standard, unmonitored care, over follow-up periods of 3 and 1 month, respectively (27, 28).

All four studies enrolled patients either during a hospitalisation for HF (ReDS, ReDS-SAFE and IMPEDANCE-HF) or during a hospitalisation or within 10 days of discharge (BMAD). All participants received wearable device readings on enrolment. Patients but not investigators were blinded to study data in the ReDS, ReDS-SAFE and IMPEDANCE-HF trials. In the BMAD trial, both investigators and patients in the monitored arm had access to the device data, whereas patients in the control arm were blinded. A total of 958 patients were included in the meta-analysis and baseline characteristics of the participants and the devices are summarised according to the individual trials in Table 2.

**Table 2:** Key baseline characteristics of patients enrolled in studies of wearable monitor-guided management of HF compared with standard care.

Baseline characteristics	Alvarez-Garcia et al	Boehmer et al	Bensimhon et al	Shochat et al
Participants (*n*)	Wearable arm: 50 Control arm: 50	Wearable arm: 249 Control arm: 245	Wearable arm: 60 Control arm: 48	Wearable arm: 128 Control arm: 128
Age (years)	Wearable arm: 67 ± 12 Control arm: 68 ± 15	Wearable arm: ≤65 years (% patients): 39.8 >65 years (% patients): 60.2 Control arm: ≤65 years (% patients): 47.3 >65 years (% patients): 52.7	Wearable arm: 73.6 Control arm: 73.6	Wearable arm: 67.5 ± 11.7 Control arm: 67.7 ± 10.4
Sex (male, %)	Wearable arm: 70 Control arm: 78	Wearable arm: 57.8 Control arm: 58.8	Wearable arm: 55 Control arm: 44	Wearable arm: 82 Control arm: 87
EF (%)	Wearable arm: 40 ± 16 Control arm: 36 ± 16	Wearable arm: • LVEF ≤40 (% patients): 42 • LVEF >40 (% patients): 55 Control arm: • LVEF ≤40 (% patients): 52 • LVEF >40 (% patients): 47	Wearable arm: • LVEF ≤40 (% patients): 63 • LVEF >40 (% patients): 37 Control arm: • LVEF ≤40 (% patients): 54 • LVEF >40 (% patients): 44	Wearable arm: 30 (25–30) Control arm: 30 (25–30)
NT-proBNP (pg/ml)	Wearable arm: 5,669 Control arm: 6,081	Wearable arm: n/r Control arm: n/r	Wearable arm: 1,200 Control arm: 1,162	Wearable arm: 2,592 ± 3,317 Control arm: 2,984 ± 3,583
NYHA Class (%)	Wearable arm: • Class III/IV: 100 Control arm: • Class III/IV: 98	Wearable arm: • I/II: 17.3 • III: 36.1 • IV: 17.3 • n/r: 29.3 Control arm: • I/II: 13.9 • III: 46.9 • IV: 11.4 • n/r: 27.8	Wearable arm: n/r Control arm: n/r	Wearable arm: • II: 48 • III: 29 • IV: 23 Control arm: • II: 47 • III: 30 • IV: 23
Prior Hospitalisation for HF (%)	Wearable arm: 100 Control arm: 100	Wearable arm: 100 Control arm: 100	Wearable arm: 100 Control arm: 100	Wearable arm: 100 Control arm: 100
Ischaemic aetiology (%)	Wearable arm: n/r Control arm: n/r	Wearable arm: 46.2 Control arm: 44.5	Wearable arm: n/r Control arm: n/r	Wearable arm: 66 Control arm: 75
Diuretics (%)	Wearable arm:(furosemide) 64 Control arm:(furosemide) 82	Wearable arm: 89.2 Control arm: 93.9	Wearable arm: 72 Control arm: 63	Wearable arm: 96 Control arm: 95
ACEi/ARB/ARNI (%)	Wearable arm: n/r Control arm: n/r	Wearable arm: 94.8 Control arm: 78.0	Wearable arm: 60 Control arm:46	Wearable arm: 96 Control arm: 96
Beta-blockers (%)	Wearable arm: n/r Control arm: n/r	Wearable arm: 81.1 Control arm: 84.5	Wearable arm: 82 Control arm: 81	Wearable arm: 92 Control arm: 90
MRA (%)	Wearable arm: n/r Control arm: n/r	Wearable arm: 25.3 Control arm: 31.4	Wearable arm: 8 Control arm: 10	Wearable arm: 65 Control arm: 58
SGLT2i (%)	Wearable arm: n/r Control arm: n/r	Wearable arm: 13.7 Control arm: 2.4	Wearable arm: - Control arm: -	Wearable arm: - Control arm: -

EF, ejection fraction; BNP, B-type natriuretic peptide; NYHA, New York Heart Association; HF, Heart Failure; ACEi, Angiotensin-converting enzyme inhibitors; ARB, Angiotensin receptor blockers; ARNI, Angiotensin receptor/neprilysin inhibitor; MRA, Mineralocorticoid receptor antagonist; SGLT2i, Sodium-Glucose Transport 2 Inhibitors.

#### Hospitalisations for HF

**Figure 1:**
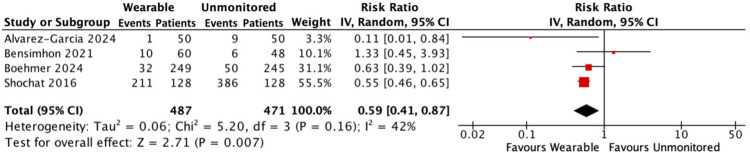
Forest plot displaying hospitalisations for HF in patients receiving wearable-guided care compared with standard care.

There were 254 hospitalisations for HF among 487 patients receiving wearable-guided care compared with 451 hospitalisations in 471 patients who received standard unmonitored treatment. Hospitalisations for HF were reduced by 41% in the wearable monitored group [RR: 0.59, 95% CI: 0.41–0.87, *p* = 0.007; moderate heterogeneity (*I*^2^ 42%)] (Figure 1).

#### Worsening HF events

**Figure 2:**
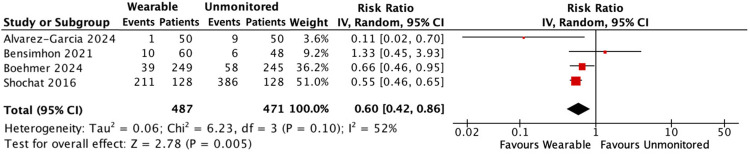
Forest plot displaying worsening HF events in patients receiving wearable-guided care compared with standard care.

261 worsening HF events occurred in the wearable monitored group compared with 459 in the standard care group. Wearable monitored care reduced the composite outcome by 40% [RR: 0.60, 95% CI: 0.42–0.86, *p* = 0.005; moderate heterogeneity (*I*^2^ 52%)] (Figure 2).

#### All-cause mortality

**Figure 3:**
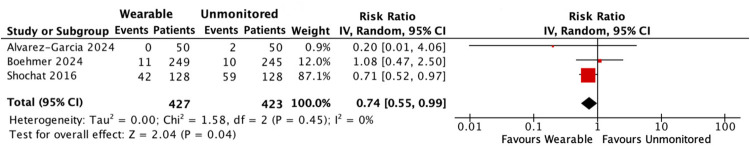
Forest plot displaying all-cause mortality in patients receiving wearable-guided care compared with standard care.

Mortality was reported in the BMAD and IMPEDANCE-HF trials. Of 427 patients in the wearable arm, 53 (12.4%) died, compared with 71 of 423 (16.8%) people in the standard care group. Wearable guided care reduced the occurrence of all-cause death by 26%. [RR: 0.74, 95% CI: 0.55–0.99, *p* = 0.04; low heterogeneity (*I*^2^ 0%)] (Figure 3).

#### Hospitalisation for HF and all-cause mortality

**Figure 4:**
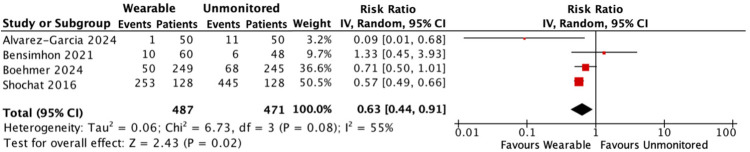
Forest plot displaying hospitalisations for HF and all-cause mortality in patients receiving wearable-guided care compared with standard care.

In a combined analysis, there were 314 events in 487 people with HF receiving treatment guided by a wearable device compared with 530 events in 471 people receiving standard care without monitoring. Hospitalisations for HF and all-cause mortality were reduced by 37% in the monitored group [RR: 0.63; 95% CI: 0.44–0.91; *p* = 0.04; moderate heterogeneity (*I*^2^ 55%)] (Figure 4).

## Discussion

This is the first meta-analysis to pool hospitalisation or mortality events from trials examining the effectiveness of wearable devices in HF. The main results support the use of wearable devices to guide care in patients with symptomatic HF irrespective of EF. Wearable device guided treatment provided a 41% reduction in the risk of hospitalisation for HF and a 26% reduction in all-cause mortality compared with standard care alone. Such substantial reductions in clinically important endpoints compare favourably alongside the benefits observed in randomised controlled trials of contemporary standards of HF care, including dapagliflozin (30% reduction in worsening HF events and 17% reduction in mortality) (29) and sacubitril-valsartan (21% reduction in hospitalisation for HF and 16% in mortality) (30). The findings of this meta-analysis also exceed the 28% reduction in hospitalisation for HF observed in the CardioMEMS Heart Sensor Allows Monitoring of Pressure to Improve Outcomes in NYHA Class III HF Patients (CHAMPION) (8) IHM trial. In the recent haemodynamic-GUIDEed management of Heart Failure (GUIDE-HF) randomised trial, neither hospitalisations for HF or mortality were reduced by IHM-guided care (31). Placed alongside CHAMPION and GUIDE-HF, there are some important differences between the IHM trials and the wearable studies included in this analysis. Firstly, CHAMPION and GUIDE-HF were multi-centre, randomised studies enrolling people with ambulatory HF (NYHA class III in CHAMPION, class II–IV in GUIDE-HF) and while prior hospitalisation for HF was an inclusion criterion in these trials, neither purposefully enrolled patients during an acute event. A formal comparison between an implanted and wearable device in a randomised trial is unlikely to ever happen. However, the lower risk option of a non-invasive wearable device that is proven to reduce clinically meaningful events would offer a sensible monitoring strategy to most clinicians.

The magnitude of relative benefit afforded to patients who were managed with wearable-based care compared with standard care alone is particularly notable given the high-risk profile of these people. In all four of the wearable studies included in this analysis, people were recruited either during a hospitalisation or within 10 days of discharge, a prognostically important period. In the three studies that reported NT-proBNP, arguably the most powerful risk predicting variable in HF, natriuretic peptide levels were markedly elevated. The rate of (re)hospitalisation in the control arms of the ReDS and IMPEDANCE-HF trials was between 50 and 94 per 100 person-years respectively, exceeding those rates observed in the same groups in the CHAMPION (68 per 100 person-years) and GUIDE-HF (49.7 per 100 person-years) trials, and considerably higher than in other contemporary trials such as the Dapagliflozin and Prevention of Adverse Outcomes in Heart Failure (DAPA-HF) trial, where the rate of hospitalisations for HF was 5–10 times lower (9.8 events per 100-person years) (29). The elevated event rate in this meta-analysis underscores how highly selected these patients were but also how effective a strategy of wearable-guided care may be to protect such a group at their most clinically vulnerable, a potentially valuable tool when transitioning care from the acute to community setting.

Several factors need to be considered to determine whether the wearable devices included in this analysis are practical monitoring options. Firstly, the duration a device was worn must be sufficient to capture meaningful amounts of data, balanced against patient comfort to ensure compliance using it. Two devices (the ReDS and LI systems) were worn in a healthcare setting for minutes at a time, minimising patient burden but healthcare providers were involved and the practicality of home use by patients was not explored. The Zoll HFMS device was used as intended within the BMAD study, for twenty-four hours a day and at-home. Data transmission rates were not reported for the BMAD trial but in wearable and IHM studies to date, adherence to data transmissions were approximately 90% of study days (8, 11). An indicator of whether a wearable device that is applied to a person's chest is likely to be adopted by patients is whether both men and women consented for the investigational study. The generalisability of wearable devices other than those included in this analysis has been reduced by near exclusive male enrolment in other studies (11). In this meta-analysis, a pooled average of 35% of participants were female, lower than real-world cohorts of people admitted to hospital with HF (32) but higher than the proportions of females recruited to other HF trials in which approximately a quarter were women (29, 30).

The included trials enrolled different populations of people with HF (patients in hospital, patients who were recently discharged, and routinely monitored ambulatory patients with HF). As the wearable devices were examined in patients in different settings and clinical status the generalisability of the meta-analysis findings are broadened. The IMPEDANCE-HF trial recruited high-risk patients with an EF ≤35%, whereas the BMAD, ReDS and ReDS SAFE trials enrolled patients with HF across the range of EF. A combined 46% of participants in the BMAD and ReDS studies had an EF >40%. The majority of patients with an EF >40% do not have an indication for an implantable device such as a defibrillator and so would not receive device-related HF diagnostics such as the COMPASS algorithm (Medtronic, Minneasota, USA). While sub-analyses of the CHAMPION study indicated that people with a preserved EF had a reduction in hospitalisation for HF, these data were limited by small numbers of patients and events (33). Among people with an EF > 40%, IHM-guided care did not reduce hospitalisations in the primary analysis in GUIDE-HF and a recent meta-analysis reported uncertainty regarding the benefits of IHM-guided care in people with an EF ≥50%1 (31, 34). Until wearable device studies report outcomes according to EF classification, it is difficult to conclude whether wearables that detect pulmonary congestion are an effective tool in caring for different HF populations. As people with preserved or milder impairment in left ventricular systolic function were participants in three of the four included studies, and given the ready application of these devices without an invasive procedure, future wearable trials should continue to actively recruit participants across the range of EF and subsequently report outcomes according to EF. Patients with both advanced and mild symptoms at baseline were recruited across the different trials. In the ReDS-SAFE HF trial, 99% of patients were NYHA class III/IV, compared with 52.5% in IMPEDANCE-HF. Rates of guideline-directed medical therapy for HF also differed across the trials, notably the use of sodium glucose co-transporter 2 (SGLT2) inhibitors and mineralocorticoid receptor antagonists (MRAs). Only the patients in BMAT study, which was conducted after the DAPA-HF (29) and Emperor-Reduced trials (35) were reported, were taking SGLT2 inhibitors at baseline. Rates of MRA use also differed substantially between studies [9.6% in ReDS (17) and 61.5% in IMPEDENCE-HF (18)]. Without individual patient-level data, which were not available for analysis, we were however unable to test for any interactions between major patient subgroups (e.g., symptoms severity, treatments, EF) and the effect of treatment guided by a wearable device or not.

This meta-analysis has several limitations. Notably, the four included studies assessed three different wearable devices for detecting pulmonary congestion, limiting direct comparability across studies. Additionally, only one study (BMAD) evaluated the device's effectiveness in a home setting, where patients used it for remote monitoring. Therefore, the feasibility of home use demonstrated in BMAD cannot be reliably extrapolated to the other devices. While both the ReDS and IMPEDANCE-HF studies were randomised controlled trials, both were conducted at single centres with expertise in the management of HF. The BMAD study was a non-randomised concurrent-control trial. Nonetheless, it is well established that patients hospitalized with HF are frequently discharged with residual congestion—a factor strongly associated with increased mortality and rehospitalization (36). Wearable technologies that detect pulmonary congestion in this vulnerable window offer an opportunity for timely intervention. Artificial intelligence (AI)-enabled devices offer a paradigm-shift in the capabilities of remote HF management. The HEARTFELT device, examined in the FOOT study, used AI to analyse 3-dimensional images of pedal oedema, presenting a novel non-invasive method to monitor for decompensation (36). The integration of AI into wearable devices and remote monitoring technologies in general is likely to develop rapidly, and as with any emerging technology should be examined in randomised clinical trials to confirm their role in the management of people with HF.

## Conclusion

HF treatment guided by wearable devices that detect pulmonary congestion reduced hospitalisations for HF and mortality in patients with HF across the range of EF. This strategy was effective in patients who had features of adverse prognosis, including a recent hospitalisation, and indicate that wearable monitoring may provide greater protection against adverse events when discharging care from the acute to home setting.

## Data Availability

The original contributions presented in the study are included in the article/Supplementary Material, further inquiries can be directed to the corresponding author.
